# Therapeutical Notes

**Published:** 1900-05-19

**Authors:** 


### THERAPEUTICAL NOTES.

### CARBOLIC ACID BURNS.

PHELPS and Powell[1] claim that the escharotic effects of carbolic acid can be entirely prevented by the use of alcohol. It was shown that when pure carbolic was poured over the hands, and they were at once dipped in strong alcohol no burns resulted. Gross put his tongue into the acid and then holding some alcohol in his mouth for 30 seconds felt no inconvenience. It was found that the same effects follow when carbolic has been taken internally. This is of special importance because, although the soluble sulphates, oil, camphor, vinegar, and white of egg have been recommended as antidotes, very little success has attended their use. A number of cases have been reported where the administration of strong alcohol was followed by recovery, and a writer who has reviewed the past history of carbolic poisoning claims that in most recorded cases of recovery alcohol was given for some reason or other. Kelly[2] saw a child of two years who had swallowed about an ounce of pure carbolic, and gave it at once six or eight teaspoonfuls of alcohol. Apomorphia was next administered to produce vomiting, and after this had acted a drachm of neat whisky was given every ten minutes till eight doses had been taken. The result of this was that the next day the child was playing about as if nothing had happened. The writer considers that if it is impossible to wash out the stomach at once with alcohol, from the absence of a stomach tube, spirit should be given immediately, and an emetic later on, to be followed by more alcohol. Adams has treated erysipelas of the hand by bathing it in pure carbolic for 30 seconds, and then washing it in alcohol. This was repeated daily, and he obtained a cure in three days.

[1] Merck's Archives, Dec. [2] Merck's Archives, Oct. [3] New York Med. Jour., Nov. 25.

### THE TREATMENT OF SCABIES.

KAPOSI lays down that the best remedies are the old Wilkinson's ointment, balsam of Peru, or the following :—

℞. Axung. porci, partes 100.
Saponis viridis, 50.
Naphtholi, 15.
Cretæ alb. pulv., 10.
Misce fiat unguentum.

This ointment should be rubbed into every fold of the skin, especially about the joints, axillæ, and pudenda, the whole body being dressed with it. Woollen clothing should be worn afterwards, and bathing forbidden for at least a week. A previous bath is not required as in the sulphur treatment.—*Landesmann Die Therapie an den Wiener Kl.*

